# A new species of *Mexicope* Hooker, 1985 (Crustacea, Isopoda) — the first record of Acanthaspidiidae Menzies, 1962 from the Mediterranean Sea

**DOI:** 10.3897/BDJ.12.e121508

**Published:** 2024-05-21

**Authors:** Torben Riehl, Katharina Ellen Schienbein, Constantine Mifsud

**Affiliations:** 1 Senckenberg Gesellschaft für Naturforschung, Frankfurt, Germany Senckenberg Gesellschaft für Naturforschung Frankfurt Germany; 2 Senckenberg Research Institute and Natural History Museum, Frankfurt am Main, Germany Senckenberg Research Institute and Natural History Museum Frankfurt am Main Germany; 3 Johann Wolfgang Goethe University Frankfurt, Frankfurt am Main, Germany Johann Wolfgang Goethe University Frankfurt Frankfurt am Main Germany; 4 Johann Wolfgang Goethe University Frankfurt, Department of Biological Sciences, Institute of Ecology, Evolution and Diversity, Frankfurt am Main, Germany Johann Wolfgang Goethe University Frankfurt, Department of Biological Sciences, Institute of Ecology, Evolution and Diversity Frankfurt am Main Germany; 5 5 Triq ir-Rghajja, Rabat, Malta 5 Triq ir-Rghajja Rabat Malta

**Keywords:** Crustacea, sp. nov., taxonomy, marine invertebrates, benthos, isopod

## Abstract

**Background:**

The marine isopod family Acanthaspidiidae Menzies, 1962 (Asellota, Janiroidea) has global distribution from shelf to hadal depth. The majority of species has been recorded from relatively deep waters and the Southern Hemisphere. To date, 36 species have been described in the family belonging to three genera: *Ianthopsis* Beddard, 1886; *Iolanthe* Beddard, 1886; and *Mexicope* Hooker, 1985.

**New information:**

Here, a new species of *Mexicope* is described from Maltese waters, adding a fourth species to the genus. It is the first member of the family reported from the Mediterranean Sea. The new species can be recognised by the unique combination of the following characters: cephalothorax with pre-ocular spine large and pointed anterolaterally, rostral projection blunt, eyes reduced; pereonal sternites each with one ventral spine; pereonite two lateral margins with single projection; pleotelson posterior apex long, projecting to approximately half of the length of the uropod protopod; pleopods I distolateral lobes projecting beyond distomedial lobes, apices curved and pointed laterally; uropod exopod length approximately 0.5 endopod length. An identification key to the species of *Mexicope* is presented and the generic diagnoses of *Ianthopsis* and *Mexicope* are compared, discussed and revised.

## Introduction

The Mediterranean Sea is often considered a marine biodiversity hot spot (Bianchi and Morri 2000, Lejeusne et al. 2010, Selig et al. 2014). It is currently undergoing a dramatic shift in faunal composition (Evans et al. 2015, Zenetos et al. 2022), yet, here we demonstrate that some of the species of this well-studied marginal land-locked sea are still unknown to science, hampering studies on biodiversity change.

In the Mediterranean Sea, 295 species of isopods have presently been recorded (Castelló et al. 2020), most of them from the western Mediterranean (Castelló 2017) suggesting sampling bias and/or the impact of the relatively strong environmental gradients. The eastern Mediterranean is, for instance, more oligotrophic in comparison to the western part (Tanhua et al. 2013). The productivity is inversely linked to the increase in temperature and salinity and, therefore, decreases from north to south and west to east. The connection to the Atlantic Ocean in the west may also have an influence on the higher diversity in the western part in relation to the eastern part as many Mediterranean species have an Atlantic origin (Danovaro et al. 1999, Pinardi et al. 2006, Coll et al. 2010).

**Figure 1.**
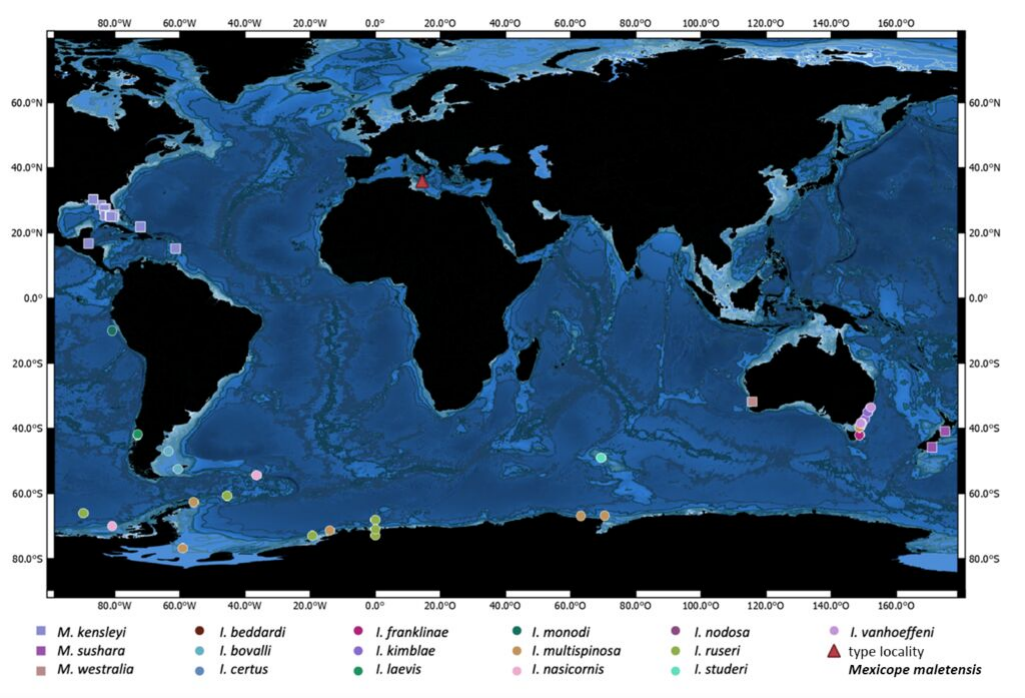
Geographic records of the three accepted species of the genus *Mexicope* and the twelve accepted species of the genus *Ianthopsis*. The red triangle symbol indicates the type locality of new *Mexicope* species.

Asellote isopods are represented by the superfamilies Aselloidea, Gnathostenetroidoidea, Janiroidea and Stenetrioidea. The family Acanthaspidiidae (Janiroidea) has not yet been reported from the Mediterranean Sea. The main distribution of this relatively small family (in terms of the number of known genera and species), established by Menzies (1962), is in deep waters and the Southern Ocean (Brandt 1991). Yet, the relatively rare genus *Mexicope* Hooker, 1985 is a good example for a widespread disjunct distribution with one of the three accepted species found in the Gulf of Mexico (Hooker 1985), one off the coast of New Zealand (Bruce 2004) and, finally, one occurs in Western Australia (Just 2001) (Fig. 1).

In its taxonomic history, Acanthaspidiidae has undergone several revisions (Bovallius 1886, Nordenstam 1933, Brandt 1991, Just 2001, Merrin and Poore 2015). The last comprehensive review by Brandt (1991) reduced the previously recognised five genera to just two, *Ianthopsis* and *Acanthaspidia*. Since then, *Iolanthe* has become the senior synonym of *Acanthaspidia* (Merrin and Poore 2015) and the genus *Mexicope*, previously designated as family incertae sedis by Hooker (1985), was added to the family (Just 2001). Since then, the family is comprised of the three genera *Ianthopsis* Beddard, 1886 (13 species), *Iolanthe* Beddard, 1886 (20 species) and *Mexicope* (3 species) (Boyko et al. 2023).

Here, a new isopod species of *Mexicope* is described from off the coast of Malta. It represents the first record of *Mexicope* and the family Acanthaspidiidae in the Mediterranean Sea and raises the number of species for the genus to four. The diagnoses of the rather similar acanthaspidiid genera *Mexicope* and *Ianthopsis* are compared, discussed and revised and an identification key to the species of *Mexicope* is presented.

## Materials and methods

### Sampling

A single specimen was collected in Maltese waters, central Mediterranean Sea, off Ġnejna Bay (Fig. 1) at a depth of 120 m. The sampling device was a custom-built rectangular dredge, 1 m wide, 20 cm high and with 500 µm mesh size. Original fixation was 90% ethanol (EtOH). Despite multiple attempts to collect more specimens of this species, only a single specimen has been collected.

### Microscopic techniques, specimen preparation and description

For the examination, dissection and voucher imaging of the specimen, a Leica M125 C stereomicroscope with motorised z-drive was used. Taxonomic drawings were made with a Leica DM2500 LED compound microscope, equipped with DIC and with a *camera lucida*. For the drawings, dissections and confocal laser scanning microscopy (cLSM), the specimen was transferred to glycerine and stained. To avoid shrinking, it was placed in a 1:1 solution of 75% ethanol and glycerine with a droplet of saturated, ethanol-based solution of Congo Red and Acid Fuchsin added (Michels 2007, Michels and Büntzow 2010). Stock solution of the dye was made according to the protocol described by Kamanli et al. (2017), but based on ethanol instead of deionised water. Over a period of at least three days, the ethanol was allowed to completely evaporate and only glycerine remained (Wilson 2008). The pencil drawings were digitised using an iPad and the Vectornator software (Linearity GmbH; Version 4.12.0). Afterwards, the digitised drawings were used to take measurements with the measurement tool in the software Foxit PDF Reader by the standards of Hessler (1970).

The cLSM scans were made using a Leica TCS SPE including a Leica DM 2500 microscope and the Leica LAS X software (Leica Application Suite X; Version 3.3.3.16958). For all scans, the 405 nm, 488 nm and 561 nm lasers were used (see Suppl. material 1 for settings). Image stacks of the multi-channel cLSM scans were edited with the software FiJi Image J (Java 1.8.0_172, 64-bit (Schindelin et al. 2012)), where the false-colour red was assigned to the 405 nm laser, green to the 488 nm laser and yellow to the 561 nm laser. Next to the false-colour assignment, the colour balance, brightness, contrast and threshold were adjusted before merging the channels. For combining the z-stack into one image, the projection types “standard deviation” or “max intensity” were used (Suppl. material 1).

For the description, terminology follows previous works on Acanthaspidiidae and other Janiroidea (Hessler 1970, Wilson 1989, Bruce 2004, Mursch 2009, Riehl and Brandt 2010). To avoid multiple repetition of the word ‘times,’ these are reported as a multiplier of an object of a telegraphic phrase to indicate the size of the subject of the phrase (see Wilson (1989), Riehl et al. (2014)). For example, ‘endopod length 2.2 width’ means ‘the length of the endopod is 2.2 times its width.’ This example is mathematically equivalent to the equation ‘L = 2.2W’. Dependent object clauses, separated off by a comma, do not repeat the subject. Characters were formatted following Sereno (2007). The map was created using the software QGIS 3.0.0. The layers GEBCO 2008 and depth contours updated in 2019 were used.

The holotype is deposited at the crustacean collection of the Senckenberg Research Institute and Natural History Museum Frankfurt, Frankfurt am Main, Germany, with the collection prefix “SMF”.

### Abbreviations

Throughout the descriptive part of this article, the following abbreviations have been used:

AI - antennula; AII - antenna; Ceph - cephalothorax; Md - mandible; MxI - maxillula; MxII - maxilla; Mxp - maxilliped; P - pereopod; Plp - pleopod; Plt - pleotelson; Prn - pereonite; UB - unequally bifid (seta); Urp - uropod.

## Taxon treatments

### Mexicope

Hooker, 1985

AC0032F9-B182-5647-B91F-5CFDC121EFD5

https://www.marinespecies.org/aphia.php?p=taxdetails&id=248964

https://www.gbif.org/species/2200137

**Composition**: *Mexicopewestralia* Just, 2001, *Mexicopesushara* Bruce, 2004 and *Mexicopemaletensis* Riehl & Schienbein, sp. nov.
Mexicope kensleyi Hooker, 1985Hooker 1985: pp. 261-265; figs. 5 and 6.

#### Description

##### Synonymy

*Mexicope* Hooker, 1985: 261. – Just (2001: 913); Bruce (2004: 2) (Hooker 1985, Just 2001, Bruce 2004).

#### Diagnosis

Cehalothorax laterally with eyestalks and developed or reduced eyes; eyestalks directed laterally or anterolaterally, with preocular spine. Pereonites 1 and 5–7 with single lateral projection; pereonites 3–4 with bifid lateral projections; pereonite 2 lateral projection single or bifid; lateral projections with apical short setae; pereonites 2–4 and 6–7 posterior projections with long setae recurving dorsally over tergites, pereonite 5 without long setae. Pereonite 7 with sternal spine. Pleotelson margins finely serrated, with spine-like, short, curved setae. Antennula length ca. 0.33 antenna length, article 1 and 2 subequal in length. Mouthparts prognathous; mandibular molar tapering or pointed with apical long setae; mandibular palp present or absent; maxilliped epipod short, reaching half way between coxobasal articulation and palp insertion. Pleopod II fringed with long setae, longest apically; stylet coiled. Uropod length equal or longer than pleotelson, peduncle and rami long and slender; rami length equal or longer than peduncle length.

##### Remarks on diagnosis

This diagnosis has been completely revised to overcome the inconsistencies and overlaps of previous diagnoses.

#### Taxon discussion

A new diagnosis for *Mexicope* is presented here because previous diagnoses of the genera *Ianthopsis* and *Mexicope* (Just 2001, Bruce 2004) show significant overlap in some character states leading to difficulties to differentiate between these genera and to classify the new species described here.

Character states that support classification as either *Ianthopsis* or *Mexicope* are the absence of dorsal tubercles on the cephalothorax, the presence of eyes on eyestalks, single lateral projections on pereonite 1, the absence of tergal spines or humps, a fringe of long setae laterally on the second pleopod basis which are longest apically, finely serrated pleotelson margins and uropod rami that are at least as long as the peduncle. The presence of a simple, bulge-shaped rostrum, the antennula length of 0.25 times antenna length, the absence of a triangular lobe on antenna article 6 and the short, curved, robust setae on the pleotelson on the one hand speak for a classification of the new species as *Ianthopsis* according to the previous diagnosis.

The classification of the new species as *Mexicope*, on the other hand, is supported by the presence of a pre-ocular spine, rows of elongate, simple setae on the posterolateral projections of pereonites 2–4 and 6–7, the presence of a sternal spine on pereonite 7, prognathous mouthparts, a strongly tapering, pointed mandibular molar with long apical setae, the lack of a mandibular palp, a short maxilliped epipod reaching only half way between the coxo-basal articulation and the palp articulation, a coiled pleopod II stylet and uropods that are as long as the pleotelson.

In summary, over ten, partially complex characters suggest the classification as *Mexicope* as opposed to only four, relatively weak characters in favour of *Ianthopsis* (see Table 1).

### Mexicope maletensis

Riehl & Schienbein sp. nov.

A64CDA30-322C-57E0-B756-170F03C53113

049ED44F-A900-47AE-A386-0126ACF7061A

#### Materials

- **Type status:** Holotype. **Occurrence:** catalogNumber: SMF 62000; individualCount: 1; sex: male; lifeStage: adult; preparations: whole animal (ETOH); occurrenceID: 58A3B6CC-79D2-5622-B2FA-A132124BDCE7; **Taxon:** scientificName: Mexicopemaletensis Riehl and Schienbein; phylum: Arthropoda; class: Malacostraca; order: Isopoda; family: Acanthaspidiidae; genus: Mexicope; specificEpithet: maletensis; scientificNameAuthorship: Riehl & Schienbein; nomenclaturalCode: ICZN; **Location:** higherGeography: Mediterranean; islandGroup: Maltese shelf; locality: off Ġnejna Bay; verbatimDepth: 120 m; verbatimLatitude: 35°56.014' N; verbatimLongitude: 14°19.325' E; decimalLatitude: 35.93356667; decimalLongitude: 14.32208333; **Identification:** identifiedBy: Torben Riehl, Katharina E. Schienbein; **Event:** samplingProtocol: custom-built rectangular dredge, 1 m wide, 20 cm high and with 500 µm mesh size; eventDate: 07-06-2017; **Record Level:** institutionCode: SMF; basisOfRecord: PreservedSpecimen

#### Description

**Figure 2.**
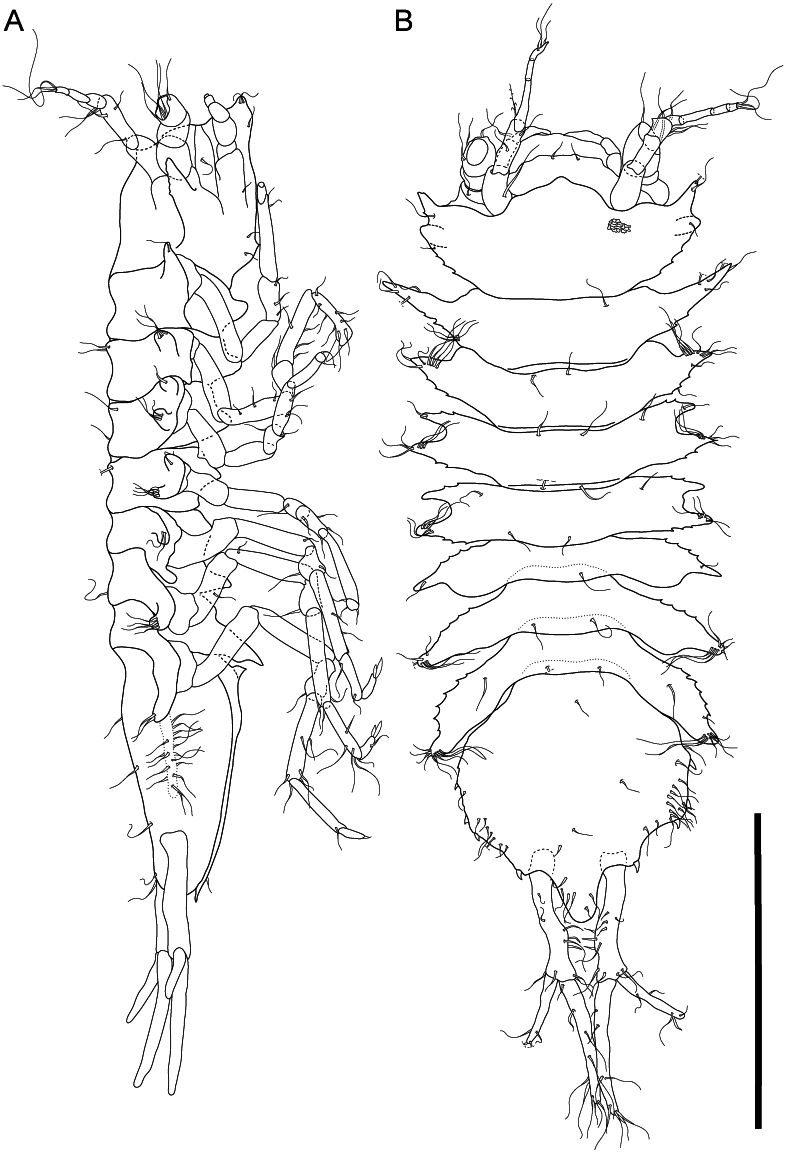
*Mexicopemaletensis* sp. nov. male holotype (SMF 62000), digitised pencil drawings of habitus: **A** habitus lateral; **B** habitus dorsal; Scale bar = 1 mm.

**Figure 3.**
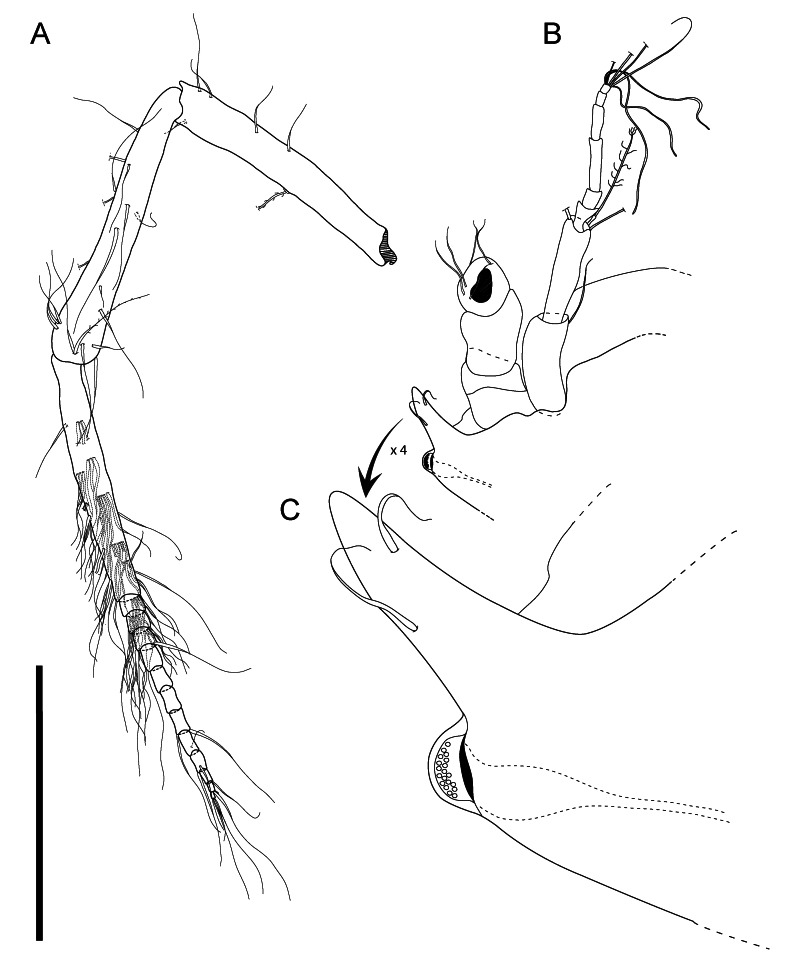
*Mexicopemaletensis* sp. nov. male holotype (SMF 62000), digitised pencil drawings of antennula and antenna: **A** antenna; **B** antennula, *in situ*. Scale bar = 0.5 mm.

**Figure 4.**
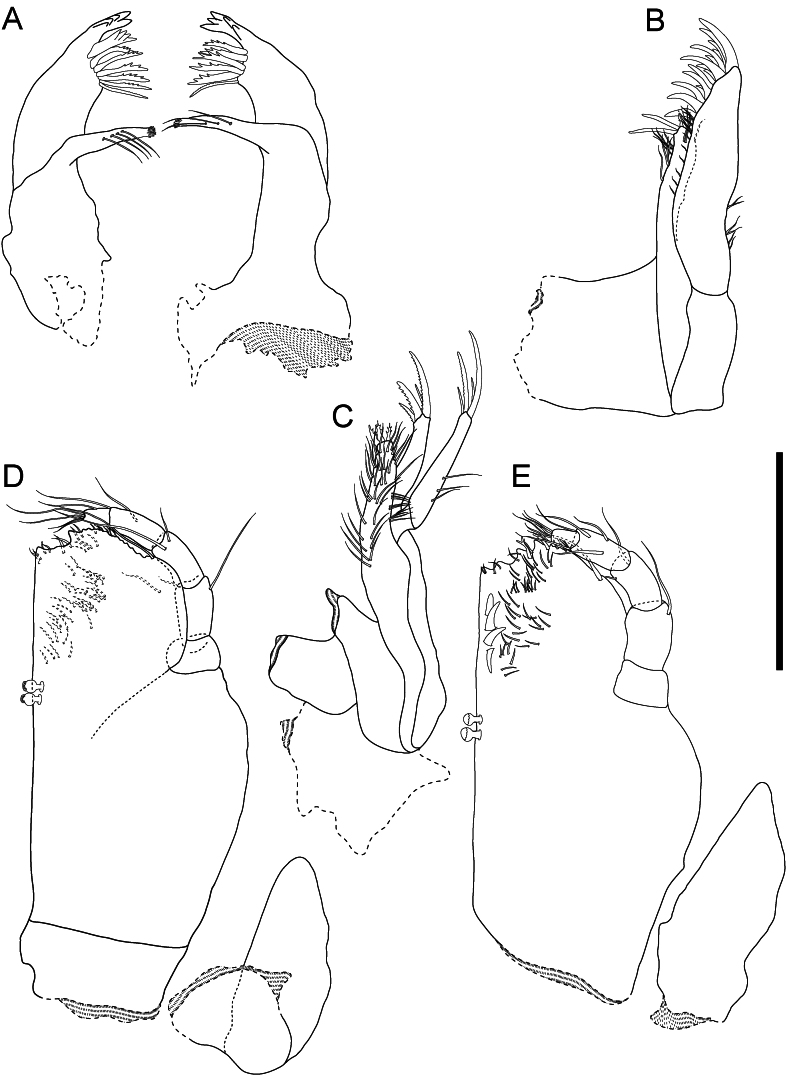
*Mexicopemaletensis* sp. nov. male holotype (SMF 62000), digitised pencil drawings of mouthparts: **A** left and right mandibles left maxilliped, dorsal aspect; **B** maxillula; **C** maxilla; **D** right mandible; **E** right maxilliped, ventral aspect. Scale bar = 250 µm.

**Body** (Fig. 2, Suppl. material 2) elongate; cuticular ornamentation small, rounded; lateral projections spine-like; length 2.5 mm; width 0.4 length; lateral margins subparallel, notched in Prn 3 and 4, spiny, pereonite 1 widest; tergites covered with scattered simple setae; relative widths of body segments: Ceph < Prn 1 > Prn2 > Prn 3 > Prn 4 < Prn 5 > Prn 6 > Prn 7 > Plt (Table 2). **Ceph** (Figs. 3–4) length 0.44 width, 0.16 body length; frontal margin rounded; frons anterior margin rounded-triangular, projecting. Eyestalks present, width 0.60 length, ocelli present. Rostrum length 0.10 Ceph length.

**Prn** (Fig. 2) tergites medially with 2–4 conspicuous long setae; sternites each with 1 spine. **Prn 1** length 0.21 width, 0.09 body length; lateral projection single, prominent, projection length 2.0 Prn2 projection length; anterolateral margin smooth; posterolateral margin serrated. **Prn 2** length 0.23 width, 0.09 body length; lateral projection single, anterolateral margin smooth, posterolateral margin serrated. **Prn 3 and 4** subsimilar, length 0.20 and 0.21 width, respectively, 0.08 body length; lateral projections bifid, anterolateral and posterolateral margins serrated. **Prn 5** length 0.15 width, 0.06 body length. **Prn 6** length 0.23 width, 0.09 body length. **Prn 7** length 0.20 width, 0.07 body length; **Prn 5-7** lateral projections single, anterolateral margins serrated, posterolateral margins smooth.

**Plt** (Fig. 2) length 1.1 width, 0.33 body length; proximal quarter narrow, lateral margins in proximal quarter convex, increasingly wide towards posterior, lateral margins posterior quarter narrowing, lateral margins convex; posterolateral margin concave at Urp insertion; posterior apex protruding, convex, width 0.23 Plt width, length 0.44 Urp protopod length; lateral margins with 4 spine-like setae; surface and margins with simple setae.

**AI** (Fig. 3B) length 0.20 body length, peduncular articles 1–2 elongated, article 3 cylindrical. Article 1 length 2.4 width, distally with 1 simple seta; article 2 length 4 width, 1.2 article 1 length, distally with 1 broom seta, 2 setae, broken, missing; article 3 length 1.0 width, 0.2 article 2 length; flagellum with 5 progressively narrower articles, length of articles 1 < 2 > 3 > 4 > 5 (Table 3); terminal article distally with 1 simple, long seta, 3 aesthetascs, 3 setae, broken, missing.

**AII** (Fig. 3A) length 0.80 body length. Article 4 distally with 5 simple setae. Article 5 longer than proximal 4 articles together, dorsal margin with 4 simple, long setae, ventral margin distally with 1 simple seta, 1 seta broken, missing. Article 6 length 1.1 article 5 length, with 19 long, simple setae, 3 broom setae. Flagellum with 12–13 articles; first article as long as carpus (peduncular article 6), length 1.4 joint length of flagellum articles 2–12, rich in aesthetascs. Aesthetascs narrow, tubular.

**Md** (Fig. 4C and D) slender, length 0.20 width, incisor of left and right Md with 5 cusps. *Laciniamobilis* with 4 cusps. Spine row of left and right Md with 6 denticulate spine-like setae, progressively increasing in length and number of denticles from distal to proximal end of spine row. *Pars molaris* slender, with 4 simple setae on left and 3 simple setae on right Md; palp absent. **MxI** (Fig. 4F) exopod length 5.7 width; margin with row of 12 simple setae, apex obliquely truncate, with 15 spine-like setae; endopod length 0.83 exopod length, length 8.3 width, medial margin with a row of 8 long, simple setae, apex narrow, with 1 serrated, spine-like and 2 simple, spine-like seta. **MxII** (Fig. 4E) both parts of exopod laterally flexed, exopod length 1.1 endopod length, lateral part slightly longer than mesial part; lateral part apex with 1 long, serrated and 3 simple, spine-like setae; mesial part apex with 3 long, serrated spine-like and 1 simple, spine-like setae; endopod slightly wider than mesial and lateral part of exopod, densely covered with long, simple setae, apically with 3 serrated, spine-like setae.

**Figure 5.**
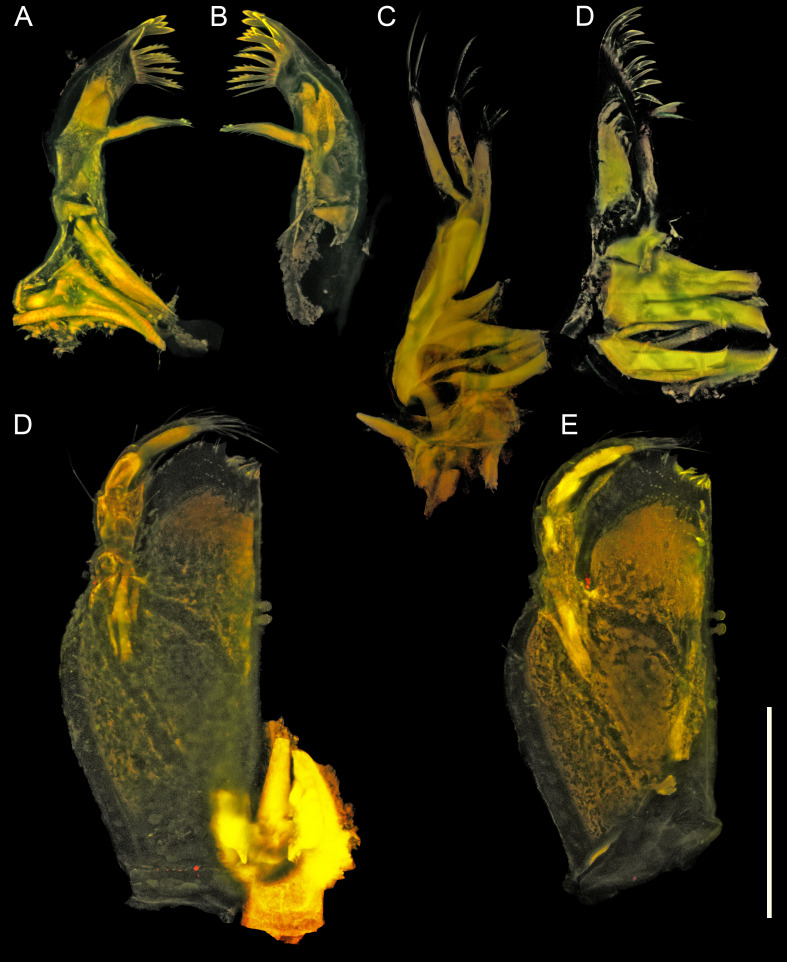
*Mexicopemaletensis* sp. nov. male holotype (SMF 62000), confocal laser scanning microscopy (cLSM) images of mouthparts: **A** left maxilliped, dorsal aspect; **B** right maxilliped, ventral aspect; **A** left mandible; **B** right mandible; **C** maxilla; **D** maxillula. Scale bar = 250 µm.

**Mxp** (Fig. 4A, B and Fig. 5) length 0.21 body length; basis and endite wide, basis length (excluding endite) 1.5 width, asetose, 2 *receptaculi*; endite ventrally densely covered with simple setae, distal margin ventrally with 2 unequally bifid spine-like setae and 1 equally bifid spine-like seta; epipodite short, reaching half way to palp articulation, acute apex, asetose, length 0.56 maxilliped length, 2.6 width; palp with 5 articles, length 0.52 maxilliped length, articles progressively narrower, merus with 2, carpus with 2, propodus with 4, dactylus with 5 sensillae.

**Figure 6.**
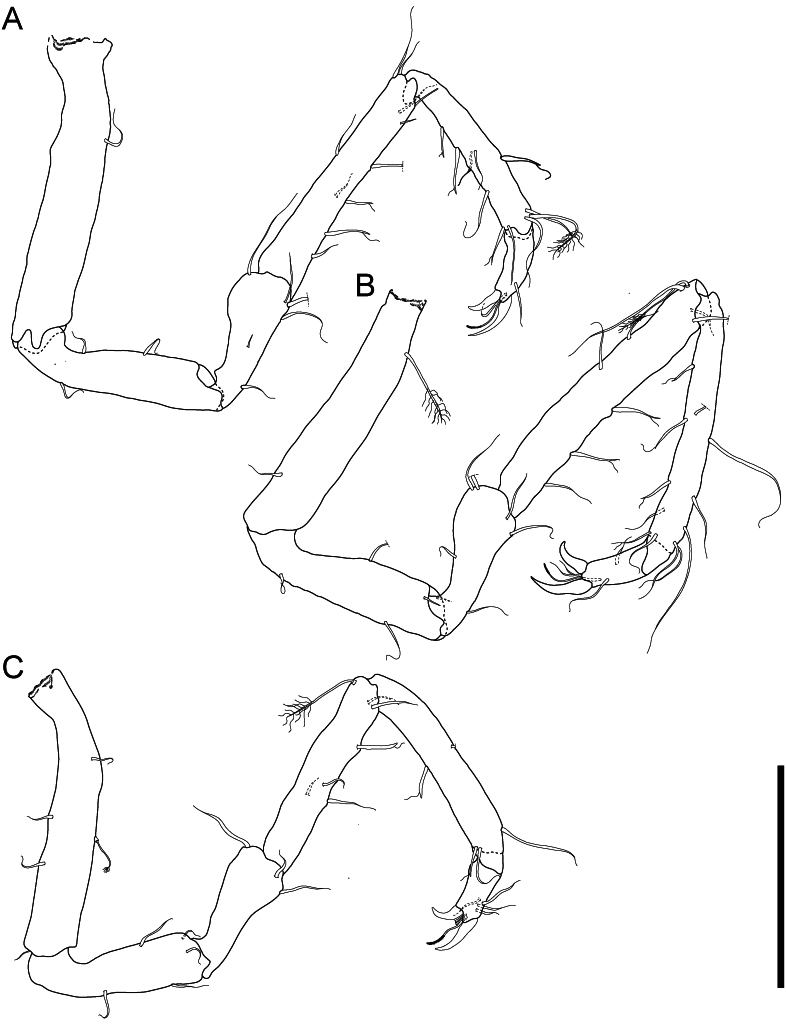
*Mexicopemaletensis* sp. nov. male holotype (SMF 62000), digitised pencil drawings of anterior pereopods in lateral view: **A** pereopod I; **B** pereopod II; **C** pereopod III. Scale bar = 250 µm.

**Figure 7.**
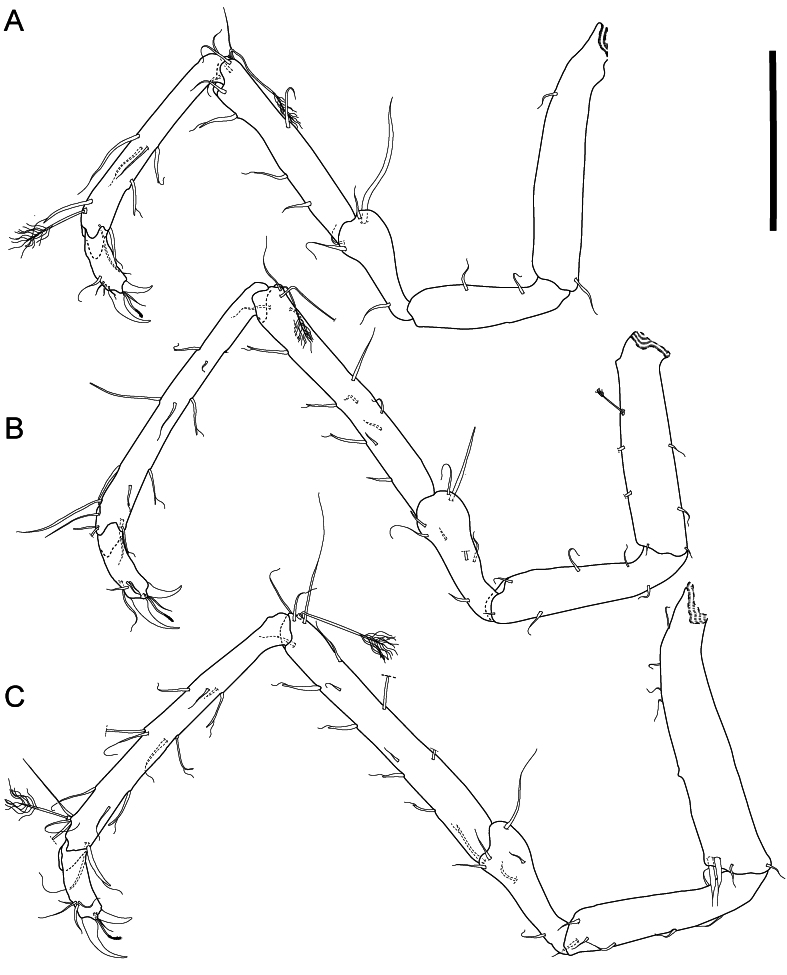
*Mexicopemaletensis* sp. nov. male holotype (SMF 62000), digitised pencil drawings of posterior pereopods in lateral view: **A** pereopod IV; **B** pereopod V; **C** pereopod VI. Scale bar 250 µm.

**Figure 8.**
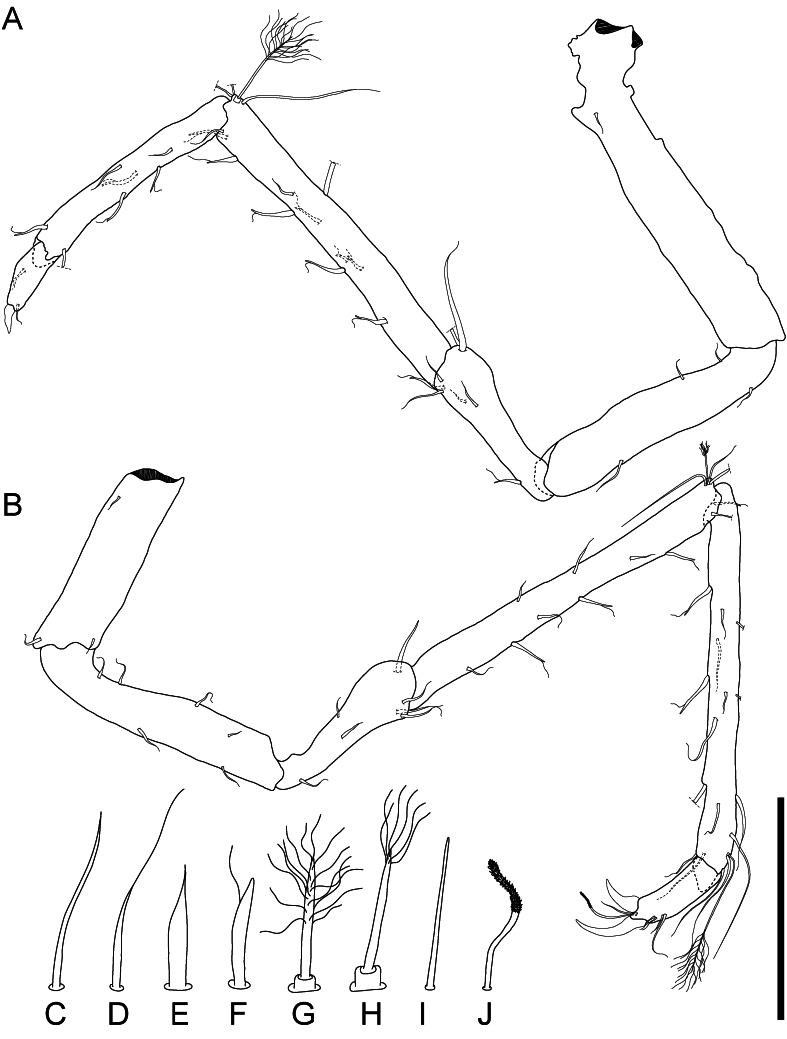
*Mexicopemaletensis* sp. nov. male holotype (SMF 62000), digitised pencil drawings of seventh pereopods in lateral view: **A** right pereopod VII; **B** left pereopod VII; **C** simple (asetulate) seta; **D** whip seta; **E** robust seta; **F** unequally bifid (sensillate) seta; **G** broom seta (distal half of shaft setulate, pappose); **H** broom seta (apically setulate, pappose); **I** simple sensilla (shaft subcylindrical, tip rounded); **J** fringed sensilla (shaft subcylindrical, distally densely covered by small scales, tip rounded). Scale bar (A–B) = 250 µm (C–I not to scale).

**P** (Figs 6, 7, 8) uniformly baculiform, ambulatory legs, length PI < II > III < IV < V < VI > VII, PII length 0.60 body length, PVI length 0.70 body length; carpi and propodi similarly long, ventral margins distodorsally with row of 1–4 robust UB setae; propodi distodorsally with 1 unequally bifid, spine-like seta; dactyli with 1 fringed sensilla between claws, distodorsal claw about half as long as dactylus, slender and acute, curved ventrally, distoventral claw length 0.80 distodorsal claw length or shorter.

**PI** (Fig. 6A) basis proximally with 1 submarginal whip seta; ischium dorsal margin with 2 whip setae, ventral margin with 1 whip seta; merus dorsal margin with 1 simple seta, ventral margin with 2 whip setae, mediodorsally with 1 simple seta; carpus dorsal margin medially with 1 whip seta, distally with 2 whip setae; ventral margin with 6 setae: 3 simple, 2 robust UB setae, 1 seta broken, missing; ventrally-submarginally with 2 simple setae; propodus dorsal margin medially with 1 robust UB seta, distally with batch of 1 broom seta and 2 whip setae; ventral margin with 3 robust UB setae and 1 whip seta; ventrally submarginally medially and laterally with, respectively, 1 simple seta, distolaterally with 1 simple seta; dactylus with 2 whip setae distodorsally.

**PII** (Fig. 6B) basis dorsal margin proximally with 1 broom seta; distoventrally with 1 whip seta; ischium dorsal margin with 1 seta, broken; ventral margin with 2 whip setae; distal margin anteriorly and posteriorly respectively with 1 simple seta; merus dorsal margin proximally with 1 whip seta, distally with 2 whip setae; ventral margin with 2 whip setae; distally submarginally with 1 simple seta; carpus dorsal margin with 4 setae: 3 whip setae, 1 broom seta; distally submarginally with 2 simple setae, laterally and medially, respectively; ventral margin with 3 setae: 1 whip, 2 UB; propodus dorsal margin with 5 whip setae; ventral margin medially with 3 UB setae, distally with 2 whip setae; distally submarginally with 1 whip seta; dactylus dorsally subdistally with 3 whip setae, ventrally submarginally with 1 whip seta.

**PIII** (Fig. 6C) basis dorsal margin with 1 whip seta and 1 broom seta; ventral margin with 3 simple setae; ischium dorsal margin with 1 simple seta, distal margin anteriorly and posteriorly, respectively, with 1 whip seta; ventral margin with 2 whip setae; merus dorsal margin distally with 1 simple seta; ventral margin distally with 1 simple seta; distal margin posteriorly with 1 whip seta; carpus dorsal margin distally with 1 broom seta; ventral margin medially with 1 whip seta, 1 robust UB seta, distal margin anteriorly and posteriorly, respectively, with 1 whip seta; medially submarginally with 2 simple setae, anteriorly and posteriorly, respectively; propodus dorsal margin medially with 1 seta, broken, missing, distally with 1 simple seta; ventral margin medially with 1 robust UB seta, distally with 1 robust UB, 1 whip seta; dactylus dorsal margin with 4 whip setae; distally anteriorly with 2 setae, broken, missing.

**PIV** (Fig. 7A) basis proximodorsally with 1 simple seta, distoventrally with 1 simple seta; ischium dorsally with 2 simple setae submarginally; merus distodorsally with 2 simple setae; ventral margin proximally with 1 simple seta, distally with simple setae; carpus dorsal margin medially with 1 simple seta, distally with 2 simple setae, 1 broom seta, 1 seta, broken, missing; ventral margin medially with 1 simple seta, 2 robust UB setae; distal margin with 3 simple setae; propodus dorsal margin with 1 whip, 1 robust UB, 1 broom seta; ventral margin with 3 robust UB setae; dactylus dorsal margin with row of 3 simple setae and 1 UB seta, distally with 1 whip seta.

**PV** (Fig. 7B) basis dorsal margin with 1 broom seta, 2 setae, broken, missing; ventral margin with 3 whip setae, distal margin with 1 simple seta; ischium dorsal and ventral margins with 2 whip setae, respectively; distal margin with 2 whip setae; merus dorsal margin distally with 1 whip seta, submarginally medially with 1 seta, broken, missing and 1 simple seta, distally with 1 simple seta; ventral margin proximally with 1 simple seta, distally with 1 whip seta and 1 simple seta; carpus dorsal margin medially with 2 whip setae, distally with 2 whip setae and 1 broom seta; ventral margin with 1 robust seta and 3 robust UB setae; distal margin posteriorly with 1 simple seta; propodus dorsal margin with 1 robust UB seta, 5 simple setae and 1 broken seta; ventral margin proximally with 1 seta, broken, missing, 3 UB setae, submarginally with 3 whip setae; dactylus distally with 5 whip setae.

**PVI** (Fig. 7C) basis dorsal margin proximally with 4 short whip setae, medially with 1 seta, broken, missing, distally with 2 robust whip setae; ventral margin distally with 1 whip seta; distal margin anteriorly with 1 whip seta; ischium dorsal margin proximally and distally with 1 simple seta, respectively; ventral margin with 3 whip setae; distal margin with 1 short whip seta anteriorly and posteriorly, respectively; merus dorsal margin distally with 1 long whip seta, medially submarginally with 1 simple seta; ventral margin proximally with 1 whip seta, distally with 1 simple seta, medially submarginally with 1 simple seta; distal margin with 1 whip seta anteriorly and 1 UB seta posteriorly; carpus dorsal margin medially with 2 broken setae, missing and 1 whip seta, distally with 3 whip setae and 1 broom seta; ventral margin with 4 robust UB setae, submarginally with 2 short whip setae; distal margin posteriorly with 1 simple seta; propodus dorsal margin with 3 simple setae, 2 long simple setae, 1 broom seta, 2 broken setae (missing), mesially with 4 simple setae, distodorsally with 1 robust UB seta, 1 broom seta; ventral margin with 1 simple seta, 4 robust UB setae, dactylus distally with 5 whip setae.

**PVII** (Fig. 8A and B) basis distodorsally with 1 short whip seta; proximoventrally with 1 simple seta and distoventrally with 1 short whip seta; ischium dorsal margin with 3 whip setae, submarginally with 1 short simple seta; ventral margin with 2 simple setae; merus dorsal margin medially with 1 short whip seta, distally with 1 long simple seta; ventral margin proximally with 1 whip seta, medially submarginally with 1 simple seta, distally with 2 long whip setae; distal margin anteriorly with 1 whip seta; carpus dorsal margin with medially with 2 whip setae, distally with 1 broom seta, 2 simple setae, 1 UB seta, 2 broken setae (missing), ventral margin with 4 robust UB setae, submarginally with 3 whip setae; propodus dorsal margin medially with 2 broken setae (missing), distally with 4 long whip setae and 1 broom seta, on right PVII with 1 UB seta; ventral margin with 4 robust UB setae, submarginally with 5 whip setae; dactylus distally with 5 whip setae.

**Figure 9.**
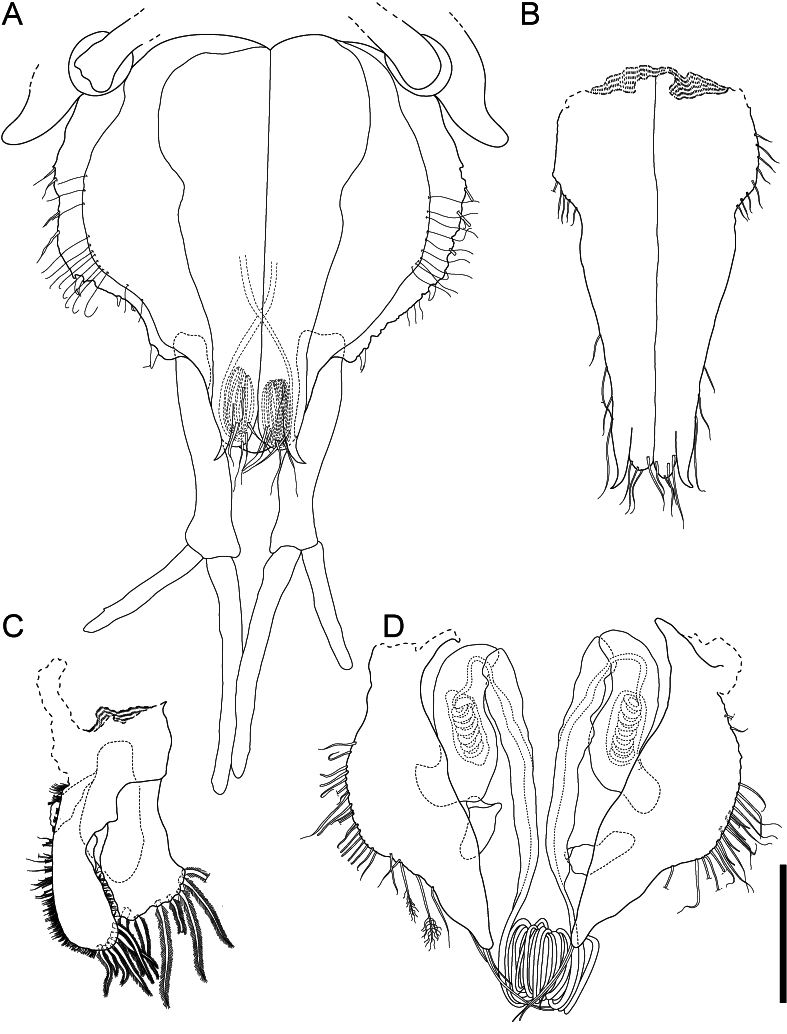
*Mexicopemaletensis* sp. nov. male holotype (SMF 62000), digitised pencil drawings pleopods: **A** pleotelson in ventral view, uropodal setae omitted; **B** pleopod I; **C** pleopods III and IV (underneath); **D** pleopod II. Scale bar = 250 µm.

**Figure 10.**
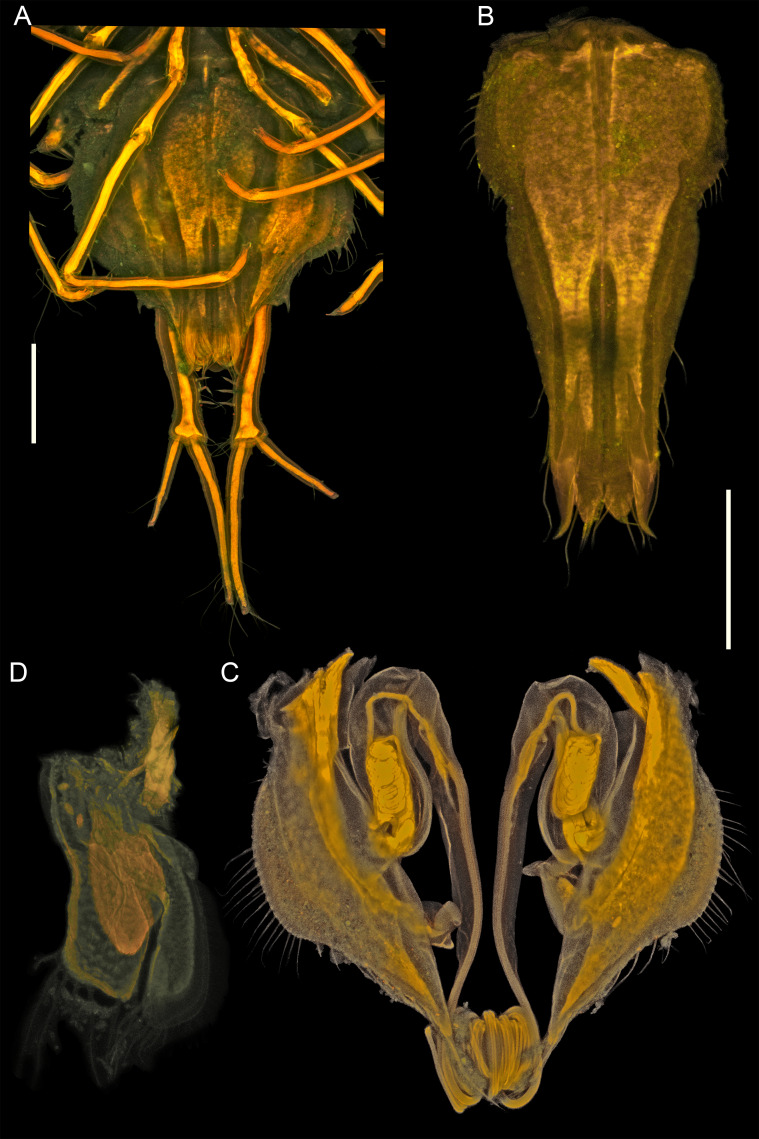
*Mexicopemaletensis* sp. nov. male holotype (SMF 62000), confocal laser scanning microscopy (cLSM) images of pleopods: **A** pleotelson in ventral view; **B** pleopod I; **C** pleopod II; **D** pleopod III and IV. Scale bar = 250 µm.

**PlpI** (Fig. 9B, Fig. 10A and B) length 2.1 width, proximally widest, with shallow lateral notch distally of proximal third, progressively narrower from notch to apex; distolateral lobes narrow, pointed and pointing caudolaterally, laterally with 3–4 long, simple setae; distomedial lobes slightly shorter and wider, apex broadly rounded, distal margin with 4 simple setae. **PlpII** (Fig. 9C and Fig. 10C) length 1.7 width, basis lateral margin mid-1/3 convex, apex narrowly rounded. Lateral margin with row of simple setae, distolateral with row of pappose setae, apex with 2 long, simple setae. Exopod asetose, length approx. 2 width, articulating at approx. 2/3 of the length of the basipodite. Endopod inserting at mid-length of basis, stylet longer than body length, distal end whip-like, coiled at least 10 times. **PlpIII** (Fig. 9D and Fig. 10D) exopod longer than endopod, distal end wide, rounded, lateral and distomesial margin densely setose, apex with 6 plumose setae; endopod distally with 7 plumose setae. **PlpIV** (Fig. 9D) exopod length and width about 0.5 endopod length and width respectively, asetose.

**Urp** (Figs 2, 9, 10) 0.33 body length, 0.96 Plt length, protopod and rami cylindrical, protopod length 8.3 width, with 6–10 simple setae scattered across article and 3 robust setae on mesial margin; endopod length 1.1 protopod length, caudally pointing parallel to longitudinal body axis, with 8–9 simple setae; endopod length 1.9 exopod length, pointing caudolaterally at an angle of ca. 45 degrees from longitudinal axis, distally with 4–5 simple setae.

#### Diagnosis

Body elongate, widest at pereonite 1. Cephalothorax rostrum bulge-shaped; eyes reduced; preocular spine pointed anterolaterally, posterolateral margin serrated. Pereonites 1–2 and 5–7 lateral projection single; pereonites 3–4 lateral projections bifid; pereonites 1–2 projections posterolateral margin serrated, pereonites 5–7 projections anterolateral margin serrated; pereonites 3–4 lateral projections anterolaterally and posterolaterally serrated; pereonites sternal spines present. Pleotelson proximal quarter narrow, gradually wider towards posterior, lateral margins in medial 2/4 concave, distal quarter narrow, tapering lateral margins convex with 4 spine-like setae on each side; posterior apex protruding. Antenna flagellomere 1 length similar to combined length of remaining 11–12 flagellomeres. Mandibular palp absent. Pleopods I lateral lobes projecting beyond medial lobes; apices pointed laterally. Uropods articulated at posterolateral pleotelson margin, extending beyond pleotelson apex; posterolateral margin concave at insertion of uropods. Uropod endopod length subsimilar protopod length, exopod length 0.50 endopod length.

#### Etymology

The name “*maletensis*” is related to the geographic location of the type locality in Malta. According to one theory about the etymology of “Malta”, the name is derived from the Phoenician “*malet*” (also spelled “*maleth*”) and means “place of refuge”, “haven” or “port”. So, “*Mexicopemaletensis*” translates into “Maltese *Mexicope*” or “*Mexicope* from Malta”.

#### Distribution

Only known from type locality. The known depth distribution of the genus *Mexicope* is extended, based on the newly-presented record. Previously ranging from 2 m to 80 m depth, *Mexicope* now has been reported from 2 m to 120 m depth. Furthermore, the distribution of the family Acanthaspidiidae is extended to include the Mediterranean Sea.

#### Taxon discussion

A comparisons between the species of *Mexicope* were based on male specimens, because only a single male specimen of *Mexicopemaletensis* sp. nov. was available for this study. While similarities to some congeners are evident in various characters, *M.maletensis* sp. nov. shows a unique combination of morphological features rendering this species overall distinct.

The assignment of the new species to the Acanthaspidiidae is based on typical family characters, such as the pointed body outline, single lateral projections on pereonites 1 and 5–7, bifid lateral projections on pereonites 3–4, antenna with a long flagellum and article 3 with exopod and cylindrical uropods (Wägele 1989, Brandt 1991, Just 2001).

Similarities between *M.westralia* and *M.maletensis* sp. nov. are: Ceph inter-antennal margin (frons) curved anteriorly; Prn dorsally with robust setae, Prn 3–4 lateral projections bifid, subequal in length, Prn 6–7 mid-sternal spine present; Plt margins finely serrate, anteriorly to Urp insertion concave, finely serrate; AII peduncle article 6 slightly longer than 5; left Md with 6 denticulate spine-like setae, molar strongly tapering; PlpI proximal 1/3 broadest, abruptly narrowing, slightly tapering towards apex; PlpII basis lateral margin mid-1/3 broadly convex with fringe of long slender setae, distal 1/3 concavely tapering to blunt point, endopod stylet long, whip-like, coiled extension; PlpIII exopod distal margin with 6 plumose setae; PlpIV exopod about half length of endopod, slender, naked; uropods about as long as Plt.

*Mexicopemaletensis* sp. nov. differs from *M.westralia* as follows: Ceph rostrum present (absent), Prn mid-sternal spine present (presence on Prn 5–7 only), Prn 2 lateral projection single (bifid), Plt proximal quarter narrow, lateral margin convex, becoming wider, lateral margins medial 1/2 concave, distal quarter narrowly tapering, lateral margins convex (widening distally, in proximal 1/3 with faintly concave margins, middle 1/3 broadly convex), Plt apical lobe twice as long as wide (half as long as wide), AII flagellum article 1 conjoint, length as long as remaining 13 short articles (half-length of 14 short articles), Md palp absent (present), right Md with 6 denticulate spine-like setae, length progressively increasing, distal to proximal denticles number increasing (with 8 such setae), MxI outer lobe with 13 spines (12 denticulate spine-like setae), Mxp basis length 1.5 width (2.5 width), PlpI dorsally without setules, without tabs (dorsal mid-surface with field of setules, more distally with 2 triangular locking tabs), PlpIII endopod with 7 plumose setae (with 6 plumose setae), Urp outer ramus length 0.50 protopod length (exopod and protopod of similar length), exopod length ca. 0.50 endopod length (ca. 0.80).

Similarities between *M.sushara* and *M.maletensis* sp. nov. are: body length 2.0 width; inter-antennal margin (frons) more or less convex, rostral spine short, pre‑ocular spines prominent, eyes laterally, stalked; Prn 1 anterolateral projections acute, curved towards anteriorly, Prn 3–4 lateral margins distinctly bifid, projections subequal in size, Prn 5–6 lateral projections directed posteriorly, Prn 2–3 and Prn 6–7 projections with anteriorly directed long dorsal setae, Prn 4 setae only on posterior lobe; Prn 7 mid-sternal spine present; AII flagellum conjoint article 1 length subequal length of remaining 12 articles; left Md lacinia mobilis with 4 cusps, molar tapering; MxII lateral and middle lobes each with 2 long and 2 short strongly serrate setae; Mxp epipodite linguiform; pereopods similar in shape, setation subequal, PI–III proportionally shorter than PIV–VII, length differences mostly caused by length variation of propodus; PlpI proximally widest, lateral margin narrowing abruptly, tapering smoothly to bilobed apex; PlpII lateral margin medially strongly convex, with dense row of simple setae, distolateral margin concave, endopod stylet long, distally coiled; PlpIII exopod distal margin with 6 plumose setae, exopod lateral margin with continuous fringe of setae; PlpIV exopod half as long as endopod, apically acute.

*Mexicopemaletensis* sp. nov. differs from *M.sushara* as follows: Ceph rostral projection blunt, barely projecting (rostrum acute, projecting and curved upwards); Plt anterior quarter narrow, lateral margin convex, becoming wider posteriorly, lateral margins medially concave, distally narrowly tapering, convex (proximally narrow, widening abruptly); Prn 2 lateral projection single (bifid); AII article 6 distodorsal triangular lobe absent (present), Md incisors with 5 cusps (with 4 cusps), right and left Md with 6 denticulate spine-like setae, length and number of denticles progressively increasing from distal to proximal (left spine row of 5 denticulate spine-like setae, 8 simple setae; right spine row with 8 denticulate spine-like setae), palp absent (present); Mxp basis length 1.5 width (2.1 width); PlpI length 2.1 width (3.9 width); PlpII length 1.7 width (2.6 width), apex with 3 long simple setae (with 6 long plumose setae), exopod apex unilobed (bilobed); PlpIII endopod distal margin with 7 plumose setae (with 6 plumose setae).

Similarities between *M.kensleyi* and the new species are: preocular spines orientated anterolaterally; eyes on stalks; Prns laterally with few setae; AI heavily setose, flagellum with 13 articles, first article longest; Md palp absent, molar process conical setiferous, left lacinia quadridentate, one distal simple spine; MxI inner ramus shorter than outer one; Mxp palp 5-segmented, segment width subequal, distal segment setose, endite broad, outer margin rounded, inner margin 2 coupling hooks, distal third with numerous setules, epipodite short, apically narrowly rounded; P propodus longest segment, merus bulbous, dactylus with two claws; PVII longest; PlpI laterally setose; PlpII protopod outer margin with long and short setae, endopod exceeding length of protopod, distally coiled; Urp elongate, biramous, peduncle length similar exopod length.

*Mexicopemaletensis* sp. nov. differs from *M.kensleyi* as follows: body length almost 2.0 width (length almost 3.0 width), eyes ventrolaterally (dorsolaterally), Prn 1 widest (Prn 3 and 4 widest); left Md distally with 5 dentate spines (with 6 spines); PlpII endopod distally coiled at least 10 times (stylet coiled once).

#### Notes

The initial difficulties we had in assigning the new species to a genus are emblematic of the currently inadequate and vague diagnoses of all three genera of Acanthaspidiidae (see also Timm et al. (2013)). With previously only three known species for the genus *Mexicope*, it is no surprise that, for each new species, a modification of the generic diagnoses was necessary and the discrimination amongst the acanthaspidiid genera was partially blurry. This development is particularly evident in *Ianthopsis* (see taxon discussion for *Ianthosis* below).

A new diagnosis for *Mexicope* is presented here because previous diagnoses of the genera *Ianthopsis* and *Mexicope* (Just 2001, Bruce 2004) show significant overlap between *Ianthopsis* and *Mexicope* in some character states leading to difficulties to differentiate between these genera and to classify the new species described here. Character states that support the classification of the new species as either *Ianthopsis* or *Mexicope* are the absence of dorsal tubercles on the cephalothorax, the presence of eyes on eyestalks, single lateral projections on pereonite 1, the absence of tergal spines or humps, a fringe of long setae laterally on the second pleopod basis which are longest apically, finely serrated pleotelson margins and uropod rami that are at least as long as the peduncle. The presence of a simple, bulge-shaped rostrum, the antennula length of 0.25 times antenna length, the absence of a triangular lobe on antenna article 6 and the short, curved, robust setae on the pleotelson on the one hand speak for a classification of the new species as *Ianthopsis* according to the previous diagnosis. The classification of the new species as *Mexicope*, on the other hand, is supported by the presence of a pre-ocular spine, rows of elongate, simple setae on the posterolateral projections of pereonites 2–4 and 6–7, the presence of a sternal spine on pereonite 7, prognathous mouthparts, a strongly tapering, pointed mandibular molar with long apical setae, the lack of a mandibular palp, a short maxilliped epipod reaching only half way between the coxo-basal articulation and the palp articulation, a coiled pleopod II stylet and uropods that are as long as the pleotelson. In summary, over ten, partially complex characters suggest the classification as *Mexicope* as opposed to only four, relatively weak characters in favour of *Ianthopsis* (see Table 1).

### Ianthopsis

Beddard, 1886

493507AF-C154-53AF-A75A-A0D99D70F189

https://www.marinespecies.org/aphia.php?p=taxdetails&id=118309

https://www.gbif.org/species/2200094

**Composition**: *Ianthopsisbeddardi* Kussakin & Vasina, 1982, *Ianthopsisbeddardi* Kussakin & Vasina, 1982, *Ianthopsiscertus* Kussakin & Vasina, 1982, *Ianthopsisfranklinae* Brandt, 1994, *Ianthopsiskimblae* Brandt, 1994, *Ianthopsislaevis* Menzies, 1962, *Ianthopsismonodi* Nordenstam, 1933, *Ianthopsismultispinosa* Vanhöffen, 1914, *Ianthopsisnasicornis* Vanhöffen, 1914, *Ianthopsisnodosa* Vanhöffen, 1914, *Ianthopsisruseri* Vanhöffen, 1914, *Ianthopsisstuderi* Kussakin & Vasina, 1982, *Ianthopsisvanhoeffeni* Just, 2001.
Ianthopsis bovalii (Studer, 1884)Beddard 1886: Museum für Naturkunde, Berlin, 5499 Synonyms: JanthebovalliiStuder 1884: 10-12; plate 1, figs. 2a–d.

#### Description

##### Synonymy

*Ianthopsis* Beddard, 1886: 15. – Nordenstam (1933: 180); Brandt (1994: 224); Just (2001: 918-919); *Janthopsis* Menzies (1962b: 83) (unjustified emendation) (Nordenstam 1933, Menzies 1962, Brandt 1994, Just 2001).

#### Diagnosis

Cephalothorax pre-ocular spine present; frontal projection (rostrum) present, rostrum shape simple (bulge-shaped) to complex (spine-like rostrum); dorsal tubercles present or absent; eyestalks present or absent. If present, eyestalk shape variable, from long stalks to low bulges; exes present or absent. Pereonites dorsal (tergal) projections absent or present, variable in shape if present (hump-shaped to spine-shaped). Pereonite 1 lateral projections either single or bifid. Pereonite 2 lateral projections bifid. Pereonites 2-4, 6-7 posterolateral projections without rows of elongate, simple setae. Pereonite 7 without sternal spine. Pleotelson with short curved robust setae.

Antenna article 6 without distal triangular lobe. Mouthparts orientation ventral or moderately prognathous. Mandibular molar shape variable, cylindrical, triturating or (rarely) tapering, without apical long setae; palp present. axilliped epipodite long, reaching to or beyond palp articulation.

Pleopod 2 stylet straight. Uropod length variable, from shorter than pleotelson to longer than pleotelson; uropodal rami shorter than or peduncle length.

#### Notes

Character states that were once considered diagnostic for *Ianthopsis* became irrelevant over time with more species being described, which is expressed in the fact that "either-or" statements survived as quasi testimonies of this development in the genus diagnosis: “cephalothorax dorsal tubercules present or absent”, “eyestalks present or absent”, “eyes present or absent”, “pereonite 1 lateral projection single or bifid”, “dorsal spines or humps on pereonites present or absent” (Table 1). This made it necessary to examine both genera in detail and to critically revise their diagnoses, which we have accomplished here.

Besides strictly diagnostic (unique) character states, the revised diagnosis includes also variable characters which are diagnostic in the genus *Mexicope* or the new species *Mexicopemaletensis*, sp. nov.

## Identification Keys

### Key to the species of *Mexicope* Hooker, 1985

**Table:** 

1	All pereonites with mid-sternal spines; pereonite 1 widest part of body; pereonite 2 lateral projection single	***Mexicopemaletensis* sp. nov.**
–	Mid-sternal spines absent or only on few pereonites; pereonite 1 narrower than some of the following segments; pereonite 2 lateral projection bifid	2
2	Pleotelson length subsimilar width; pleotelson posterior apex length exceeding width; mandibular palp absent	***M.kensleyi* Hooker, 1985**
–	Pleotelson length smaller width; pleotelson posterior apex length smaller width; mandibular palp present	3
3	Cephalothorax rostrum absent; pereonite 1 narrower than pereonite 2; pereonites 5–7 mid-sternal spine present	***M.westralia* Just, 2001**
–	Cephalothorax rostrum present; pereonite 1 wider than pereonite 2; pereonites 5–7 mid-sternal spine absent	***M.sushara* Bruce, 2004**

## Discussion

Initial difficulties in assigning the new species to a genus reflect the inadequate and vague diagnoses of Acanthaspidiidae genera (see also Timm et al. (2013)). With only three known species in the genus *Mexicope*, modifications to generic diagnoses were inevitable for each new species, leading to blurred discrimination amongst the genera. This trend is particularly evident in *Ianthopsis*, where once-diagnostic character states became irrelevant over time, as seen in Table 1. Similarly, *Mexicope*'s diagnosis weakened over time, complicating clear differentiation between *Ianthopsis* and *Mexicope*. Consequently, the new species *Mexicopemaletensis* sp. nov. initially lacked a definitive genus assignment, prompting a detailed examination and critical revision of both genera's diagnoses, which we have undertaken here.

As more new species of Acanthaspidiidae are discovered, further family revisions will be needed for clear genus distinctions. Though not delving into a full family revision here, certain characteristics are earmarked for future systematic analyses: rostrum (presence, shape, length), eyes (presence, orientation, ommatidia count), eyestalks (length, width, orientation, association with pre-ocular spine), pereonite 1 lateral projection (shape, length, orientation, serration, setation), mid-sternal spines (presence, size, shape), male pleopod II stylet (shape) and uropods (relative lengths, widths, protopod, rami orientation, setation).

For future analyses of *Mexicope*, attention should be paid to rostrum presence and shape, eyestalks, antenna II, mandibular palp, pereonite 2 lateral projection and male pleopod II stylet shape. Additionally, phylogenetics could aid in species and genus differentiation by shedding light on character evolution and illuminate family origins. Expanding the sparse molecular data currently available (Raupach et al. 2004, Raupach and Wägele 2006, Raupach et al. 2009) and integrating these data for phylogenetic assessment hold significant promise.

## Supplementary Material

XML Treatment for Mexicope

XML Treatment for Mexicope maletensis

XML Treatment for Ianthopsis

41A637EB-1228-5B0F-83C6-623422A46CAF 10.3897/BDJ.12.e121508.suppl1 Supplementary material 1 Confocal laser scanning microscope (cLSM) and FiJi ImageJ settings used for imaging *Mexicopemalitensis* sp. nov. Data type word document Brief description Overview of the specimen examined by cLSM with information on the laser line, laser intensity, detection range, detector gain, lens, frames and FiJi ImageJ projections type for respective figures. The lens used was an ACS APO 10x/0.30 DRY and ACS APO 20x/0.60 IMM. The number of frames was set to 1.5 times the suggested system optimised number. Scan speed was 400 Hz, the scan direction was unidirectional. The pinhole aperture was 94.3 µm. The specimen was stained with Acid Fuchsin and Congo Red. PMT = photomultiplier tube; CH1–CH3 = detection channels 1–3; STD = Standard Deviation; MI = Max Intensity. All objects were embedded in glycerine. File: oo_985834.docx https://binary.pensoft.net/file/985834 Torben Riehl, Katharina Ellen Schienbein, Constantine Migfsud

A5EC336A-2725-5121-96D0-DB52350ACD5F 10.3897/BDJ.12.e121508.suppl2 Supplementary material 2 Microscopic images of *Mexicopemaletensis* sp. nov. male holotype Data type Figure Brief description *Mexicopemaletensis* sp. nov. male holotype (SMF 62000): A habitus, lateral; B habitus, dorsal; C habitus, ventral. Scale bar = 1 mm. File: oo_1011605.png https://binary.pensoft.net/file/1011605 Torben Riehl, Katharina Schienbein

## Figures and Tables

**Table 1.** Comparison of diagnostic characters of *Ianthopsis* (Just 2001) and *Mexicope* (Just 2001, Bruce 2004) with those of the new species *Mexicopemaletensis* sp. nov. Similarities/overlaps are highlighted in grey, entirely identical character states mentioned in the aforementioned diagnoses were omitted.

**Character**	**Character states *Ianthopsis* Beddard, 1886**	**Character states *Mexicope* Hooker, 1985**	**Character states *Mexicopemalitensis* sp. nov.**
cephalothorax frontal projection (rostrum)	present	absent	present
cephalothorax rostrum shape	simple (bulge) / complex (spine-like rostrum)	spine-like rostrum in *M.sushara*	simple (bulge)
cephalothorax dorsal tubercles presence	present / absent	present / absent	absent
cephalothorax eyestalks	present / absent	present	present
cephalothorax eyestalks shape	long stalks / low bulges	long stalks / low bulges	low bulges
cephalothorax eyes presence	present / absent	present	present
cephalon preocular spine	absent	present	present
pereonite 1 lateral projection	single / bifid	single	single
pereonite 2 lateral projection	bifid	single / bifid	single
pereonites 2-4, 6-7 posterolateral projections rows of elongate, simple setae	absent	present	present
pereonites dorsal spines/humps	present / absent	absent	absent
pereonite 7 sternal spine	absent	present	present
antenna article 6 distal triangular lobe	absent	present / absent	absent
mouthparts orientation	ventral / moderately prognathous	prognathous	prognathous
mandibular molar shape	cylindrical, triturating / rarely tapering	strongly tapering / pointed	strongly tapering / pointed
mandibular molar apical long setae presence	absent	present	present
mandibular palp presence	present	present / absent	absent
maxilliped epipodite length versus maxilliped basis length	long (reaching to or beyond palp articulation)	short (reaching half way to palp articulation)	short (reaching half way to palp articulation)
pleopod 2 stylet shape	straight	coiled	coiled
pleotelson setation	short curved robust setae	absent	short curved robust setae
uropods length versus pleotelson length	shorter than pleotelson / longer than pleotelson	as long as / longer than pleotelson	as long as pleotelson
uropod rami length versus peduncle length	shorter than / similar peduncle length	similar / longer than peduncle	similar / longer than peduncle

**Table 2.** *Mexicopemaletensis* sp. nov. male holotype (SMF 62000) relative pereonite lengths. L = length; L/W = length-width ratio.

**Proportion**	**Pereonite 1**	**Pereonite 2**	**Pereonite 3**	**Pereonite 4**	**Pereonite 5**	**Pereonite 6**	**Pereonite 7**
**L/W**	0.21	0.23	0.20	0.21	0.15	0.22	0.20
**L/body L**	0.09	0.09	0.08	0.08	0.06	0.09	0.07

**Table 3.** *Mexicopemaletensis* sp. nov. male holotype (SMF 62000) antennula relative article lengths. L = length; L/W = length-width ratio.

**Proportion**	**Article 1**	**Article 2**	**Article 3**	**Article 4**	**Article 5**	**Article 6**	**Article 7**	**Article 8**
**L/W**	2.4	3.8	1.3	1.3	5.0	3.0	1.0	2.0
**L/article 2 L**	0.9	1.0	0.3	0.2	0.5	0.3	0.1	0.1
