# Supplementary material for: A new species of Mexicope Hooker, 1985 (Crustacea, Isopoda) — the first record of Acanthaspidiidae Menzies, 1962 from the Mediterranean Sea
Source: Biodivers Data J. 2024 May 21;12:e121508. doi: 10.3897/BDJ.12.e121508 (PMC11134053; doi:10.3897/BDJ.12.e121508)
Supplement: Supplementary material 1 — Confocal laser scanning microscope (cLSM) and FiJi ImageJ settings used for imaging Mexicopemalitensis sp. nov. [file bdj-12-e121508-s001.docx]

**Supplementary Table S1:** Confocal laser scanning microscope (cLSM) and FiJi ImageJ settings used for imaging *Mexicope malitensis* **n. sp.** Overview of the specimen examined by cLSM with information on the laser line, laser intensity, detection range, detector gain, lens, frames and FiJi ImageJ projections type for respective figures. The lens used was an ACS APO 10x/0.30 DRY and ACS APO 20x/0.60 IMM. The number of frames was set to 1.5 times the suggested system optimized number. Scan speed was 400 Hz, the scan direction was unidirectional. The pinhole aperture was 94.3 µm. The specimen was stained with Acid Fuchsin and Congo Red. PMT = photomultiplier tube; CH1–CH3 = detection channels 1–3; STD = Standard Deviation; MI = Max Intensity. All objects were embedded in glycerin.

7

| Figure | Laser line $\left[ \mathbf{nm} \right]$ / Intensity $\left[ \boldsymbol{\%} \right]$ | Laser line $\left[ \mathbf{nm} \right]$ / Intensity $\left[ \boldsymbol{\%} \right]$ | Laser line $\left[ \mathbf{nm} \right]$ / Intensity $\left[ \boldsymbol{\%} \right]$ | Detection range PMT $\left[ \mathbf{nm} \right]$/ gain $\left[ \mathbf{V} \right]$ | Detection range PMT $\left[ \mathbf{nm} \right]$/ gain $\left[ \mathbf{V} \right]$ | Detection range PMT $\left[ \mathbf{nm} \right]$/ gain $\left[ \mathbf{V} \right]$ | Lens | Frames | FiJi ImageJ projection type |
| --- | --- | --- | --- | --- | --- | --- | --- | --- | --- |
| Maxilliped (right) | CH1: 405 / 8.87 | CH2: 488 / 20.2 | CH3: 561 / 46.02 | 418 – 551  630 | 493 – 643  750 | 567 – 716  794 | 20x | 80 | MI |
| Maxilliped (left) | CH1: 405 / 8.87 | CH2: 488 / 20.2 | CH3: 561 / 46.02 | 418 – 551  651 | 493 – 643  756 | 567 – 716  794 | 20x | 95 | MI |
| Mandible (right) | CH1: 405 / 8.87 | CH2: 488 / 20.2 | CH3: 561 / 46.02 | 418 – 551  662 | 493 – 643  756 | 567 – 716  794 | 20x | 95 | MI |
| Mandible (left) | CH1: 405 / 8.57 | CH2: 488 / 20.2 | CH3: 561 / 46.02 | 418 – 551  651 | 493 – 643  756 | 567 – 716  794 | 20x | 100 | MI |
| Maxillula | CH1: 405 / 8.87 | CH2: 488 / 20.2 | CH3: 561 / 46.02 | 418 – 551  625 | 493 – 643  756 | 567 – 716  794 | 20x | 60 | MI |
| Maxilla | CH1: 405 / 8.87 | CH2: 488 / 20.2 | CH3: 561 / 46.02 | 418 – 551  621 | 493 – 643  756 | 567 – 716  794 | 20x | 65 | MI |
| Pleotelson (ventral) | CH1: 405 / 8.27 | CH2: 488 / 20.2 | CH3: 561 / 46.02 | 418 – 551  709 | 493 – 643  755 | 567 – 716  794 | 10x | 165 | STD |
| Pleopod I | CH1: 405 / 8.57 | CH2: 488 / 20.2 | CH3: 561 / 46.02 | 418 – 551  666 | 493 – 643  756 | 567 – 716  794 | 10x | 100 | STD |
| Pleopod II | CH1: 405 / 8.77 | CH2: 488 / 20.2 | CH3: 561 / 46.02 | 418 – 551  674 | 493 – 643  756 | 567 – 716  794 | 10x | 70 | MI |
| Pleopod III + IV | CH1: 405 / 8.77 | CH2: 488 / 20.2 | CH3: 561 / 46.02 | 418 – 551  644 | 493 – 643  756 | 567 – 716  794 | 10x | 40 | MI |
