# Supplementary figures and images for: A new species of Mexicope Hooker, 1985 (Crustacea, Isopoda) — the first record of Acanthaspidiidae Menzies, 1962 from the Mediterranean Sea
Source: Biodivers Data J. 2024 May 21;12:e121508. doi: 10.3897/BDJ.12.e121508 (PMC11134053; doi:10.3897/BDJ.12.e121508)

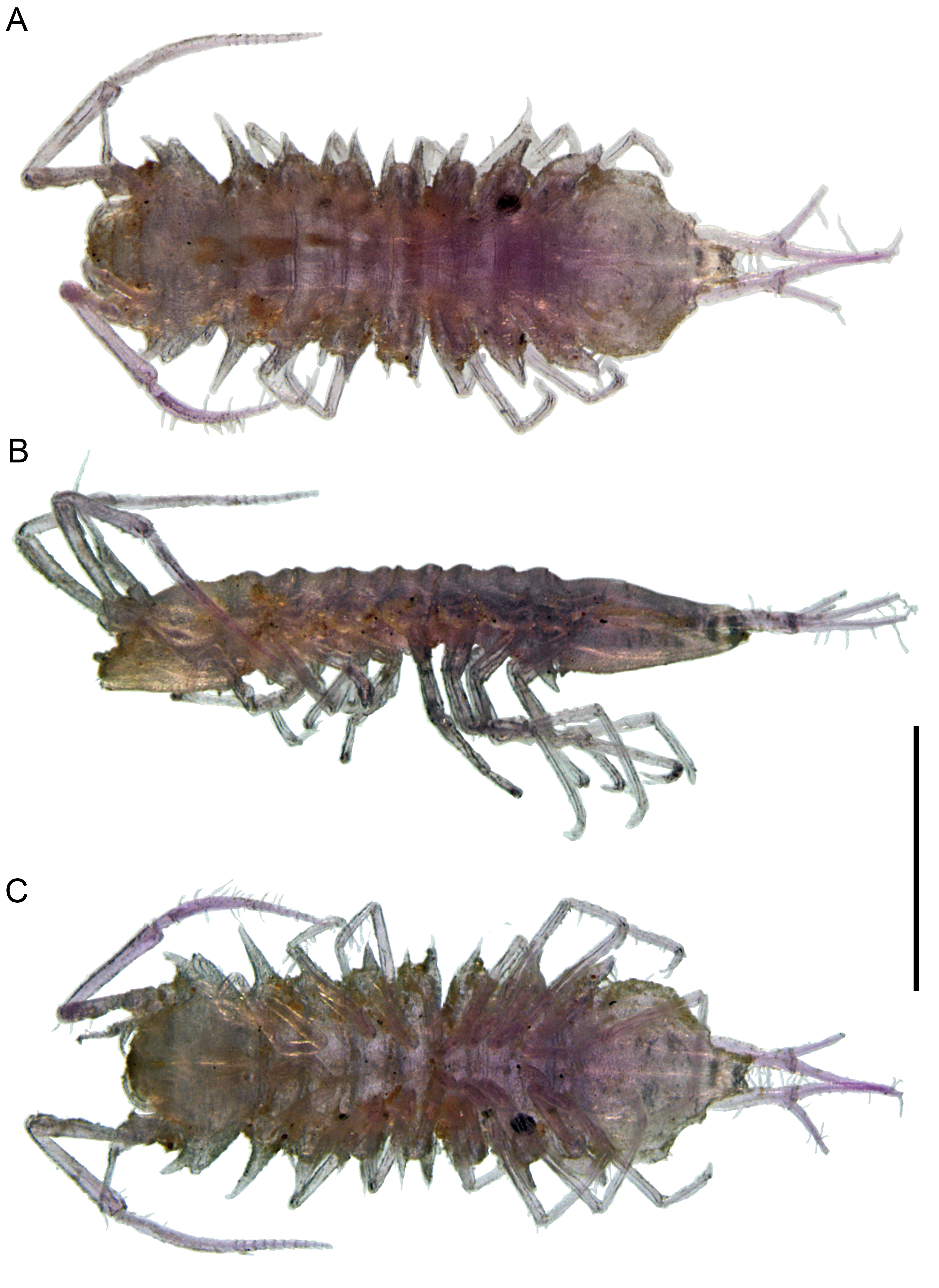

Supplement: Supplementary material 2 — Microscopic images of Mexicopemaletensis sp. nov. male holotype [file bdj-12-e121508-s002.png]
